# Optimization of Crude Oil and PAHs Degradation by* Stenotrophomonas rhizophila* KX082814 Strain through Response Surface Methodology Using Box-Behnken Design

**DOI:** 10.1155/2016/4769542

**Published:** 2016-12-28

**Authors:** Praveen Kumar Siddalingappa Virupakshappa, Manjunatha Bukkambudhi Krishnaswamy, Gaurav Mishra, Mohammed Ameenuddin Mehkri

**Affiliations:** Department of Biotechnology, The Oxford College of Engineering, Bengaluru 560068, India

## Abstract

The present paper describes the process optimization study for crude oil degradation which is a continuation of our earlier work on hydrocarbon degradation study of the isolate* Stenotrophomonas rhizophila *(PM-1) with GenBank accession number* KX082814*. Response Surface Methodology with Box-Behnken Design was used to optimize the process wherein temperature, pH, salinity, and inoculum size (at three levels) were used as independent variables and Total Petroleum Hydrocarbon, Biological Oxygen Demand, and Chemical Oxygen Demand of crude oil and PAHs as dependent variables (response). The statistical analysis, via ANOVA, showed coefficient of determination *R* ^2^ as 0.7678 with statistically significant *P* value 0.0163 fitting in second-order quadratic regression model for crude oil removal. The predicted optimum parameters, namely, temperature, pH, salinity, and inoculum size, were found to be 32.5°C, 9, 12.5, and 12.5 mL, respectively. At this optimum condition, the observed and predicted PAHs and crude oil removal were found to be 71.82% and 79.53% in validation experiments, respectively. The % TPH results correlate with GC/MS studies, BOD, COD, and TPC. The validation of numerical optimization was done through GC/MS studies and % removal of crude oil.

## 1. Introduction

Bioremediation is an ecologically acceptable technology that employs the use of microorganisms to efficiently degrade pollutants [1]. The strain* Stenotrophomonas rhizophila* (PM-1) showed potential crude oil and PAHs degrading ability in our earlier report [2]. In the present study, an attempt has been made to optimize the process of bioremediation through Response Surface Methodology (RSM), which is a reliable and powerful tool for modelling and optimization of bioremediation processes [1].

In RSM, the Box-Behnken Design is having the maximum efficiency for an experiment involving three factors and three levels; further, the number of experiments conducted for this is much less compared to a central composite design. Box-Behnken Designs always have three levels for each factor and are purpose built to fit a quadratic model [3, 4]. The Box-Behnken Design does not have runs at the extreme combinations of all the factors but compensates by having better prediction precision in the centre of the factor space. While a run or two can be botched in these designs the accuracy of the observations in the remaining runs is critical to the dependability of the model. Categoric factors can be added to these designs; however, the design is duplicated for every categoric treatment combination. It is well established that, in biological treatment processes, various operational parameters such as the level of temperature, salinity concentration, inoculum size, and pH directly influence the bacterial degradation performance of PAHs and crude oil [5]. Thus, to make the process more efficient, faster, and practically applicable, studies on the effect of each factor on the bacterial degradation of PAHs and crude oil appear essential [6]. Hence in this context, the present study was designed with an attempt to optimize cultural (pH, temperature, dose of inoculum, and salinity concentration) factors using conventional (one-factor-at-a-time) and statistical Response Surface Methodologies (RSMs) for degradation of crude oil and PAHs by the strain* Stenotrophomonas rhizophila *PM-1.

The present work discusses the use of Box-Behnken Design approach to plan the experiments for crude oil degradation with an overall objective of optimizing the process to degrade the crude oil.

## 2. Materials and Method

### 2.1. Soil Sampling and Isolation of Bacteria

The soil samples were collected from different localities of Western Ghats of Karnataka State, covering the oil spilled areas and the hydrocarbon degrading bacteria were isolated using R_2_A media followed by serial dilution using standard protocols.

### 2.2. Preliminary Degradation Studies, PAHs, and Crude Oil Utilization Studies

The preliminary degradation was studied using redox indicator 2,6-dichlorophenolindophenol (DCPIP) [7]. The 2% PAHs and crude oil utilization by the bacterial isolate using Bacto Bushnell Hass broth was studied using decanol, hexadecane, toluene, dodecane, engine oil, benzene, octane, oleic acid, and naphthalene as sole carbon source and checked for utilization.

### 2.3. Experimental Design

The Box-Behnken factorial experimental design had employed four independent variables, namely, temperature (25, 30, 35, and 40°C), pH (5, 7, 9, and 11), salinity (5, 10, 15, and 20), and inoculum size (5, 10, 15, and 20 ml) as mentioned in Table 1. Each of the independent variables was studied at three levels (1, 0, and +1), with 29 experimental runs and one control. The levels were selected based on the results of experimental designs as shown in Table 2. The full strength media with 2% crude oil/PAHs act as a control. The statistical software Design-Expert® 10 (Stat-Ease, Inc., Minneapolis, MN, USA) was used to evaluate the analysis of variance (*P* < 0.05) to determine the significance of each term in the fitted equations and to estimate the goodness of fit in each case. In order to visualise the relationship between the experimental variables and responses, 3D plots are generated from the models. The optimum variables are obtained from the response surface.

### 2.4. Extraction of Residual Oil and Total Petroleum Hydrocarbon (TPH) Analysis

Based on the preliminary degradation results, the potential bacterial strains were selected and checked for the utilization capability of the crude oil. The isolates were inoculated into the conical flask containing Bacto Bushnell Hass broth in artificial sea water along with 2% crude oil and dextrose as additional carbon source [2]. The flask was monitored at regular intervals of time up to 15 days. The flasks were observed for any changes in the physical nature of the oil.

### 2.5. Biological Oxygen Demand (BOD) and Chemical Oxygen Demand (COD) Analysis

In order to assess the rate of degradation and PAHs utilization by the isolate* S. rhizophila* KX082814, the BOD and COD analysis were performed by following standard methods (APHA, 2001, and IS-3025).

### 2.6. GC/MS Analysis to Validate the RSM Design

The GC/MS analysis was performed using a MS-5973 spectrometer coupled to a Hewlett-Packard Model 6890 and GC equipped with a cool-on-column inlet and capillary direct interface. The instrument conditions were the following: capillary column HP-1MS, 60 m × 0.2 mm; helium column flow 1 ml/min; pressure 18.5 psi; and split ratio 20 : 1. The initial temperature was 70°C and kept for 5 minutes with a temperature ramp of 14°C per minute and final temperature of 280°C was kept for 10 minutes with total run time 3024 minutes. A solvent delay was employed in order to prolong detector lifetime from 0 to 4.5 minutes. The solvent front reached the detector in 4.0 minutes and initial analyte retention time was approximately 7 minutes, so there was no loss of resolution due to initial solvent delay. The solvent used in all analyses was mixture of hexanes.

### 2.7. 16s rRNA Sequencing and NCBI Gene Bank Deposition

Bacterial identification was carried through 16s rRNA sequencing. Bacterial genomic DNA was isolated using the Insta GeneTM Matrix genomic DNA isolation kit Catalog # 732-6030. Using 16s rRNA universal primers gene fragment was amplified using MJ Research PTC-225 Peltier Thermal Cycler. The sequence obtained is deposited in the NCBI gene bank using the tool Sequin.

## 3. Results and Discussions

The present study was undertaken to examine the cumulative effect of four different parameters on degradation of crude oil and PAHs. The second-order polynomial coefficient for each term of the equation was determined through multiple regression analysis using the Design-Expert v.10. The experimental design and response for each trail were mentioned in Table 3. Maximum degradation was observed in case of run number 10 followed by 11, 17, and 7, where % degradation was found to be 70.25, 69.05, 68.54, and 67.84, respectively. It was noticed that the similar results were observed in case of runs number 10 and 11; this may be due to the identical experimental conditions, whereas in case of runs number 7 and 17, although the experimental conditions are different, the results show the highest rate of degradation was almost equal to the runs number 10 and 11. Hence according to the model, the runs number 10 and 11 as standard optimized conditions were selected as the optimum conditions to enhance the degradation. Further, the obtained optimized conditions from the RSM-BBD were confirmed and validated by experimental studies at standard conditions.

The obtained results were checked for their fitness to the model and obtained data was illustrated. The predicted and actual values for the model, Cook's distance, and studentized residuals illustrate the normal distribution and constant variance of the residuals; according to Table 4 and Figure 4, there were no points that were potentially powerful due to their location in the factor indicating the goodness of fit.

By the model, *F*-value of 3.31 implies that the model is significant (Table 5). Values of “Prob > *F*” less than 0.0500 indicate model terms are significant. In this case *A*, *C*, *A* ^2^, and *C* ^2^ are significant model terms as values greater than 0.1000 indicate the model terms are not significant. The lack of fit *F*-value of 0.19 implies the lack of fit is not significantly relative to the pure error. There is a 98.50% chance that a lack of fit *F*-value this large could occur due to noise. Nonsignificant lack of fit is good and we want the model to fit. The “Pred *R* ^2^” of 0.3240 is not as close to the “Adj *R* ^2^” of 0.5355 as one might normally expect; that is, the difference is more than 0.2. This may indicate a large block effect or a possible problem with our model and/or data. Things to consider are model reduction, response transformation, outliers, and so forth. All empirical models should be tested by confirmation runs. “Adeq Precision” measures the signal to noise ratio.

*Final Equation in Terms of Coded Factors*

(1) Total  Petroleum  Hydrocarbon  %TPH=+64.76−2.67∗A−1.34∗B−2.53∗C−0.013∗D−0.42∗AB+2.04∗AC−3.77∗AD−0.48∗BC−2.14∗BD−2.89∗CD−5.98∗A2+0.81∗B2−3.96∗C2−0.52∗D2,

where *A*, *B*, *C*, and *D* are the coded values of the test variables (Table 6), temperature (°C), pH, salinity, and inoculum size (ml), respectively. The equation in terms of coded factors can be used to make predictions about the response for given levels of each factor. By default, the high levels of the factors are coded as +1 and the low levels of the factors are coded as −1. The coded equation is useful for identifying the relative impact of the factors by comparing the factor coefficients.

*Final Equation in Terms of Actual Factors*

(2) Total  Petroleum  Hydrocarbon  TPH=−71.82338+7.08541∗Temperature+0.17307∗pH+1.05685∗Salinity+3.81374∗Inoculum  Size−0.018667∗Temperature∗pH+0.036222∗Temperature∗Salinity−0.067067∗Temperature∗Inoculum  Size−0.021222∗pH∗Salinity−0.095222∗pH∗Inoculum  Size−0.051422∗Salinity∗Inoculum  Size−0.10625∗Temperature2+0.090120∗pH2−0.070359∗Salinity2−9.24741E−003∗Inoculum  size2

**Figure 1:**
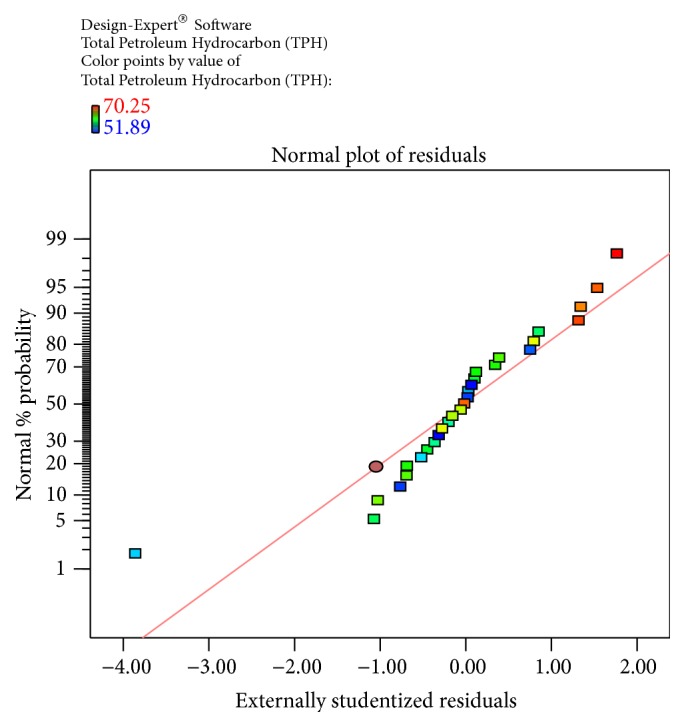
The Diagnostic Plots obtained by the Box-Behnken Design to evaluate the normal plot residuals using the normal % probability versus externally studentized residuals by the bacterial isolate* S. rhizophila KX082814* for its crude oil degradation ability.

**Figure 2 fig2:**
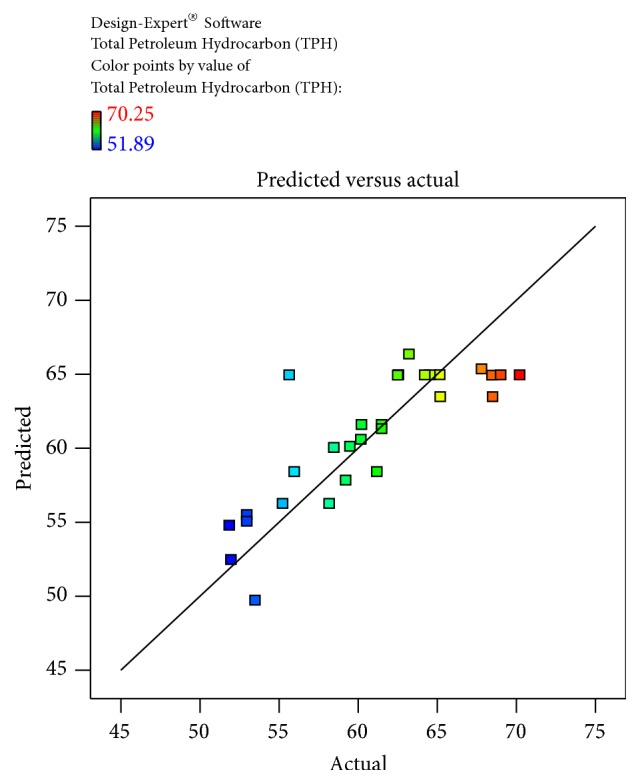
Predicted versus actual plot obtained by the Box-Behnken Design based on the % TPH of crude oil and PAHs degradation using the bacterial isolate* S. rhizophila KX082814.*

**Figure 3 fig3:**
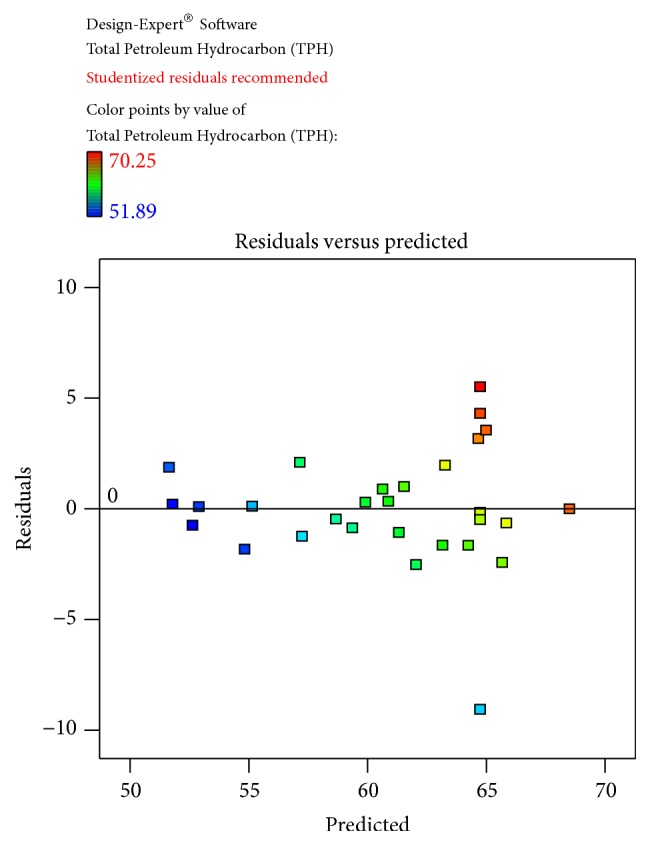
The Diagnostic Plots showing the recommended studentized residuals obtained by the Box-Behnken Design using the residual versus predicted by the bacterial isolate* S. rhizophila KX082814* for its crude oil degradation ability.

**Figure 4 fig4:**
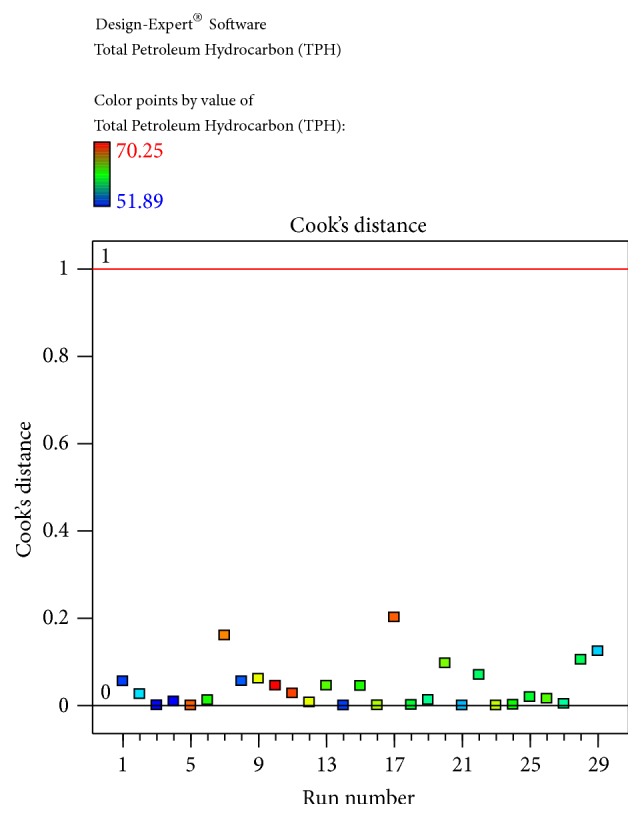
The Diagnostic Plots showing Cook's distance plot obtained by the Box-Behnken Design using Cook's distance versus run number by the bacterial isolate* S. rhizophila KX082814* for its crude oil degradation ability.

**Figure 5:**
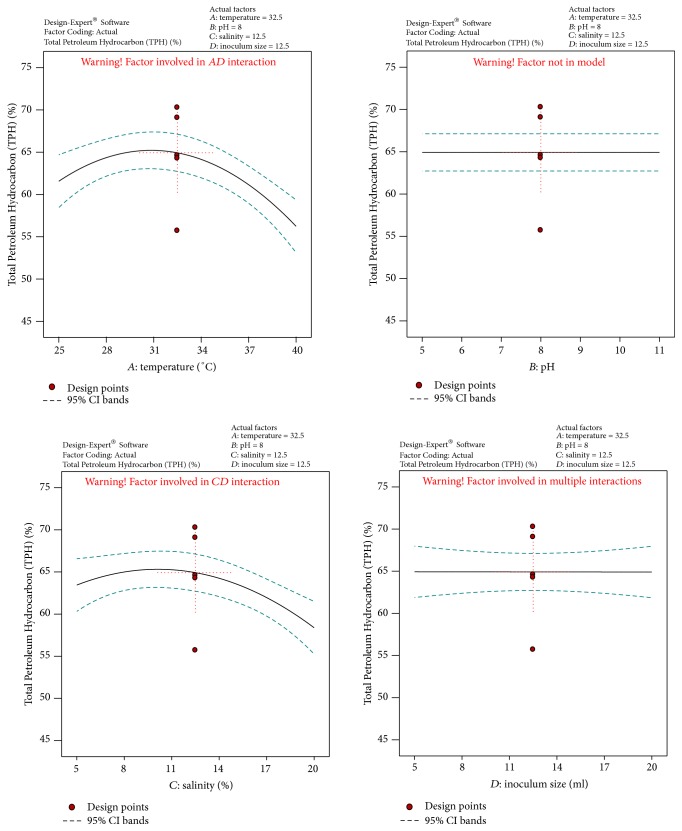
The Diagnostic Plots obtained by the Box-Behnken Design showing the interactions among factors that influence crude oil and PAHs degradation by the bacterial isolate* S. rhizophila KX082814* for its crude oil degradation ability.

The equation in terms of actual factors can be used to make predictions about the response for given levels of each factor. Here, the levels should be specified in the original units for each factor. This equation should not be used to determine the relative impact of each factor because the coefficients are scaled to accommodate the units of each factor and the intercept is not at the centre of the design space. The Diagnostic Plots are as shown in Figures 1 [Fig fig2] [Fig fig3] [Fig fig4]–5.  Figure 1 shows the normal probability plot of the studentized residuals to check for normality of residuals.  Figure 2 shows studentized residuals versus predicted values to check for constant error in the design.  Figure 3 shows externally studentized residuals to look for outliers, that is, influential values of the design. Figure 4 shows Box-Cox plot for power transformations, where Cook's distance and studentized residual illustrate the normal distribution and constant variance of the residuals and Figure 5 shows the interactions among factors that influence crude oil and PAHs degradation by the isolate* S. rhizophila* (PM-1) KX082814.

**Figure 6:**
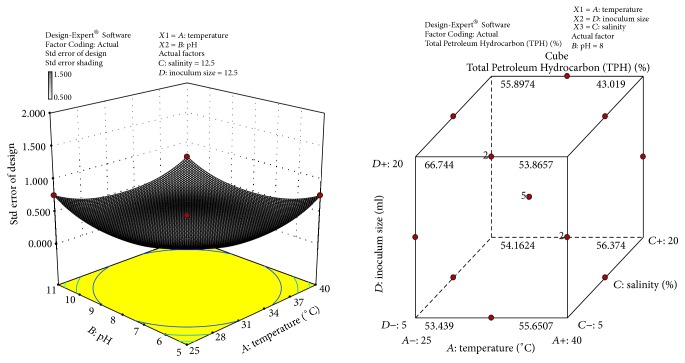
3D response surface plot and Cube representation showing the standard error of design and interaction effect of factors that influence crude oil and PAHs degradation by the bacterial isolate* S. rhizophila KX082814.*

**Figure 7 fig7:**
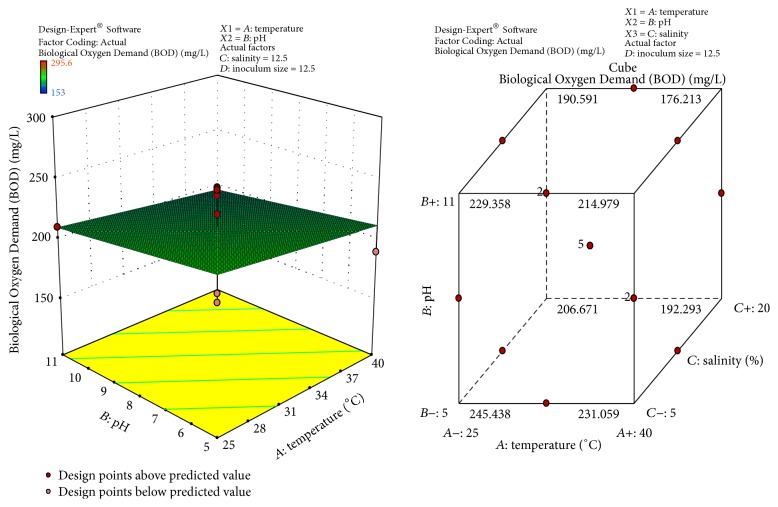
3D response surface plot and Cube representation showing the interaction effect of factors that influence BOD of crude oil and PAHs degradation by the bacterial isolate* S. rhizophila KX082814.*

**Figure 8 fig8:**
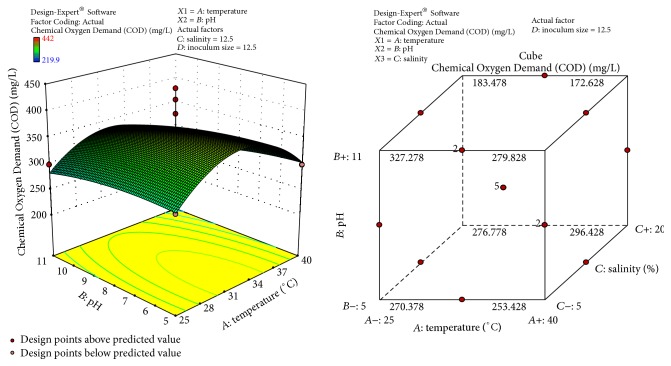
3D response surface plot and Cube representation showing the interaction effect of factors that influence COD of crude oil and PAHs degradation by the bacterial isolate* S. rhizophila KX082814.*

**Figure 9:**
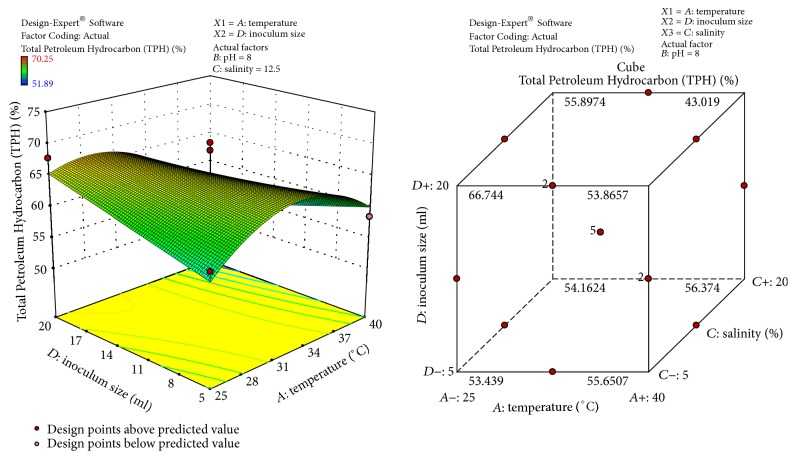
3D response surface plot and Cube representation showing the interaction effect of factors that influence % TPH of crude oil and PAHs degradation by the bacterial isolate* S. rhizophila KX082814.*

The response surface curves show the relative effects of two variables, by keeping the other variable at fixed level, on crude oil degradation. The 3D plots and cubic designs are shown in Figures 6 [Fig fig7] [Fig fig8]–9. The result obtained shows that pH of 8, temperature of 32.5°C, inoculum size of 12.5 ml, and salinity concentration of 12.5 were the best conditions to obtain maximum degradation of crude oil and PAHs using the bacterial isolate* S. rhizophila* KX082814. The optimal values for the variables as predicted by the RSM were found within the Box-Behnken Design region.

**Figure 10:**
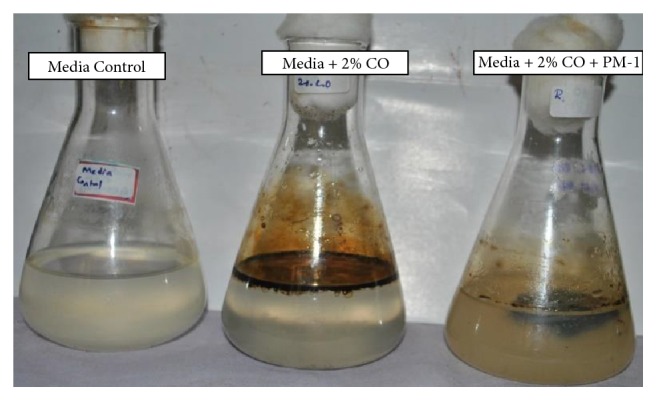
Validation of degradation using predicted optimum conditions (at temperature of 32.5°C, pH of 9, 12.5 of salinity, and 12.5 ml of inoculum size). From left the Media Control (FSM-ASW), Positive Control (FSM-ASW + 2% CO), and Test (FSM-ASW + 2%CO + PM-1 bacterial isolate) on 15th day of incubation time.

**Figure 11:**
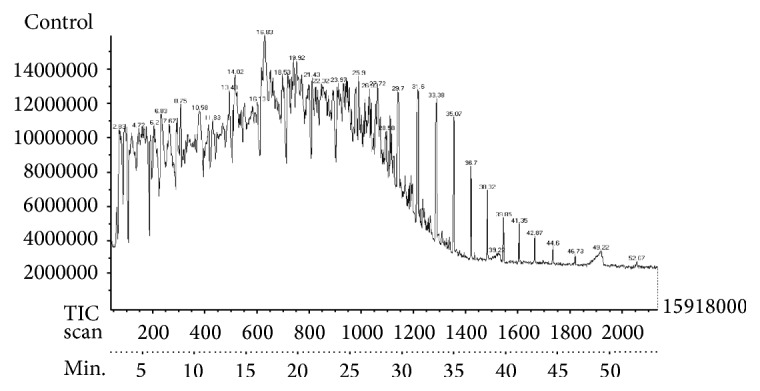
GC/MS chromatogram for 2% crude oil at 15th day of incubation shows persistence of total 40 different hydrocarbon components.

**Figure 12:**
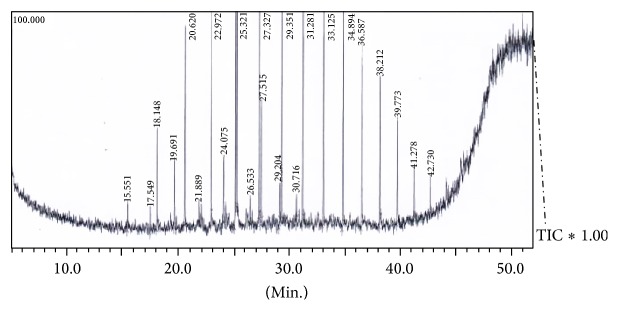
GC/MS chromatogram for 2% crude oil at 15th day of incubation shows persistence of total 23 different hydrocarbon components after being treated with the bacterial isolate* S. rhizophila* KX082814 on 15th day.

*Validation of Optimization Process*. Validation experiments were conducted in triplicate to determine the performance of* S. rhizophila* KX082814 and reproducibility of the results by evaluating the level of BOD, COD, TPC, % TPH, and GC/MS studies at the optimum favourable conditions through Box-Behnken Design and RSM (Table 7; Figure 10). The results showed 79.53 ± 2.5% of crude oil removal efficiency. The percentage error between the predicted and actual values was found to be 0.75%. The GC/MS study clearly indicates the removal of majority of both aromatic and aliphatic hydrocarbons present in the crude oil (Figures 11 and 12; Table 8). A total of 42 different hydrocarbon components were observed in control sample, where after the bioremediation treatment with the isolate* S. rhizophila (KX082814),* it was noticed that only 20 hydrocarbon components remained in the test sample after 15th day. It was observed that majority of the components in the control samples are completely degraded and some peaks are converted to simpler hydrocarbon moieties. From the results the noticeable difference in the degradation process was clearly observed.

## 4. Conclusion

This study reveals the bioremediation of crude oil and PAHs utilization by the isolate* Stenotrophomonas rhizophila* (PM-1) with gene bank accession number KX082814 could be achieved up to 79.53 ± 2.5% by maintaining the optimum parameters, namely, temperature, pH, salinity, and inoculum size. The GC/MS studies also indicate that the degradation of majority of the hydrocarbon components, the statistical analyses, and the closeness of the experimental results and model predictions show the reliability of the regression model.

## Figures and Tables

**Table 1:** Experimental range and the variables showing the limiting factors.

Name	Unit	Type	Low	High
Temperature	°C	Factor	25	40
pH		Factor	5	11
Salinity		Factor	5	20
Inoculum size	ml	Factor	5	20
Total Petroleum Hydrocarbon (TPH)	%	Response		
Biological Oxygen Demand (BOD)	mg/L	Response		
Chemical Oxygen Demand (COD)	mg/L	Response		

**Table 2:** Full-factorial Box-Behnken Design levels for the four independent variables showing total of 29 sets of experimentation work.

Std	Run	Temp (°C)	pH	Salinity	Inoculum size (ml)
8	1	32.5	8	20	20
16	2	32.5	11	20	12.5
12	3	40	8	12.5	20
18	4	40	8	5	12.5
23	5	32.5	5	12.5	20
6	6	32.5	8	20	5
11	7	25	8	12.5	20
20	8	40	8	20	12.5
14	9	32.5	11	5	12.5
29	10	32.5	8	12.5	12.5
25	11	32.5	8	12.5	12.5
22	12	32.5	11	12.5	5
21	13	32.5	5	12.5	5
19	14	25	8	20	12.5
1	15	25	5	12.5	12.5
28	16	32.5	8	12.5	12.5
13	17	32.5	5	5	12.5
5	18	32.5	8	5	5
10	19	40	8	12.5	5
7	20	32.5	8	5	20
4	21	40	11	12.5	12.5
9	22	25	8	12.5	5
27	23	32.5	8	12.5	12.5
15	24	32.5	5	20	12.5
3	25	25	11	12.5	12.5
24	26	32.5	11	12.5	20
2	27	40	5	12.5	12.5
17	28	25	8	5	12.5
26	29	32.5	8	12.5	12.5

**Table 3:** Showing the % TPH, BOD, and COD results of 29 experimental designs using *S. rhizophila *KX082814 when crude oil was used as sole carbon source on 15th day of incubation period.

Std	Run	Temp (°C)	pH	Salinity	Inoculum size (ml)	% TPH	BOD (mg/l)	COD (mg/l)
8	1	32.5	8	20	20	53	153	226.8
16	2	32.5	11	20	12.5	56	189	245.6
12	3	40	8	12.5	20	52	178	286.5
18	4	40	8	5	12.5	51.89	196.5	258
23	5	32.5	5	12.5	20	68.5	221	356
6	6	32.5	8	20	5	61.53	216	341
11	7	25	8	12.5	20	67.84	256	382
20	8	40	8	20	12.5	53.52	188	256
14	9	32.5	11	5	12.5	65.23	225	358
29	10	32.5	8	12.5	12.5	70.25	241	442
25	11	32.5	8	12.5	12.5	69.05	221	395
22	12	32.5	11	12.5	5	65.2	198	305
21	13	32.5	5	12.5	5	62.59	225.6	298.5
19	14	25	8	20	12.5	53	184.2	219.9
1	15	25	5	12.5	12.5	61.52	205	298.5
28	16	32.5	8	12.5	12.5	64.25	236.5	336.8
13	17	32.5	5	5	12.5	68.54	295.6	358
5	18	32.5	8	5	5	60.21	258	356.5
10	19	40	8	12.5	5	58.5	187.5	265
7	20	32.5	8	5	20	63.25	225	398.5
4	21	40	11	12.5	12.5	55.26	198	268
9	22	25	8	12.5	5	59.25	189.65	258
27	23	32.5	8	12.5	12.5	64.58	242.6	421.2
15	24	32.5	5	20	12.5	61.22	215.8	395.8
3	25	25	11	12.5	12.5	60.25	210.5	298.5
24	26	32.5	11	12.5	20	62.54	235.6	329.5
2	27	40	5	12.5	12.5	58.21	189.58	298.5
17	28	25	8	5	12.5	59.52	178.5	258.5
26	29	32.5	8	12.5	12.5	55.68	153.8	221.35

**Table 4:** Diagnostics case statistics, experimental design, and results of Box-Behnken Design for crude oil and PAHs degradation.

Run order	Actual value	Predicted value	Residual	Leverage	Internally studentized residual	Externally studentized residual	Cook's distance	Influence on fitted value DFFITS	Standard order
15	61.52	63.19	−1.67	0.583	−0.694	−0.681	0.045	−0.805	1
27	58.21	58.69	−0.48	0.583	−0.201	−0.194	0.004	−0.229	2
25	60.25	61.34	−1.09	0.583	−0.455	−0.442	0.019	−0.523	3
21	55.26	55.17	0.092	0.583	0.038	0.037	0.000	0.044	4
18	60.21	59.94	0.27	0.583	0.115	0.111	0.001	0.131	5
6	61.53	60.66	0.87	0.583	0.363	0.352	0.012	0.416	6
20	63.25	65.70	−2.45	0.583	−1.019	−1.021	0.097	−1.208	7
1	53.00	54.85	−1.85	0.583	−0.770	−0.759	0.055	−0.898	8
22	59.25	57.17	2.08	0.583	0.866	0.858	0.070	1.015	9
19	58.50	59.38	−0.88	0.583	−0.368	−0.357	0.013	−0.422	10
7	67.84	64.69	3.15	0.583	1.312	1.350	0.161	1.597	11
3	52.00	51.81	0.19	0.583	0.078	0.075	0.001	0.089	12
17	68.54	65.01	3.53	0.583	1.471	1.542	0.202	1.824	13
9	65.23	63.28	1.95	0.583	0.812	0.801	0.062	0.948	14
24	61.22	60.90	0.32	0.583	0.132	0.127	0.002	0.150	15
2	56.00	57.27	−1.27	0.583	−0.527	−0.513	0.026	−0.607	16
28	59.52	62.06	−2.54	0.583	−1.060	−1.065	0.105	−1.260	17
4	51.89	52.65	−0.76	0.583	−0.319	−0.308	0.009	−0.365	18
14	53.00	52.93	0.074	0.583	0.031	0.030	0.000	0.035	19
8	53.52	51.67	1.85	0.583	0.772	0.760	0.056	0.899	20
13	62.59	64.26	−1.67	0.583	−0.698	−0.685	0.045	−0.810	21
12	65.20	65.87	−0.67	0.583	−0.278	−0.268	0.007	−0.317	22
5	68.50	68.52	−0.025	0.583	−0.010	−0.010	0.000	−0.012	23
26	62.54	61.56	0.98	0.583	0.410	0.398	0.016	0.470	24
11	69.05	64.76	4.29	0.200	1.290	1.324	0.028	0.662	25
29	55.68	64.76	−9.08	0.200	−2.732	−3.852	0.124	−1.926	26
23	64.58	64.76	−0.18	0.200	−0.055	−0.053	0.000	−0.026	27
16	64.25	64.76	−0.51	0.200	−0.154	−0.149	0.000	−0.074	28
10	70.25	64.76	5.49	0.200	1.651	1.773	0.045	0.886	29

**Table 5:** Analysis of variances using ANOVA for response surface by quadratic model.

Source	Sum of squares	df	Mean square	*F*-value	*P* value, Prob > *F*	
Model	639.46	14	45.68	*3.31*	*0.0163*	*Significant*
*A*-temperature	85.33	1	85.33	6.18	0.0262	
*B*-pH	21.60	1	21.60	1.56	0.2316	
*C*-salinity	76.86	1	76.86	5.56	0.0334	
*D*-inoculum Size	1.875*E* − 003	1	1.875*E* − 003	1.357*E* − 004	0.9909	
*AB*	0.71	1	0.71	0.051	0.8245	
*AC*	16.61	1	16.61	1.20	0.2914	
*AD*	56.93	1	56.93	4.12	0.0618	
*BC*	0.91	1	0.91	0.066	0.8010	
*BD*	18.36	1	18.36	1.33	0.2683	
*CD*	33.47	1	33.47	2.42	0.1419	
*A* ^2^	231.68	1	231.68	16.77	0.0011	
*B* ^2^	4.27	1	4.27	0.31	0.5871	
*C* ^2^	101.60	1	101.60	7.35	0.0169	
*D* ^2^	1.76	1	1.76	0.13	0.7268	
Residual	193.42	14	13.82			
Lack of fit	62.13	10	6.21	0.19	0.9850	Not significant
Pure error	131.28	4	32.82			
Cor total	832.88	28				

**Table 6:** Coded values of the test variables.

Factor	Coefficient	df	Standard	95% CI	95% CI	VIF
	estimate		error	Low	High	
Intercept	64.76	1	1.66	61.20	68.33	
*A*-temperature	−2.67	1	1.07	−4.97	−0.37	1.00
*B*-pH	−1.34	1	1.07	−3.64	0.96	1.00
*C*-salinity	−2.53	1	1.07	−4.83	−0.23	1.00
*D*-inoculum Size	−0.013	1	1.07	−2.31	2.29	1.00
*AB*	−0.42	1	1.86	−4.41	3.57	1.00
*AC*	2.04	1	1.86	−1.95	6.02	1.00
*AD*	−3.77	1	1.86	−7.76	0.21	1.00
*BC*	−0.48	1	1.86	−4.46	3.51	1.00
*BD*	−2.14	1	1.86	−6.13	1.84	1.00
*CD*	−2.89	1	1.86	−6.88	1.09	1.00
*A* ^2^	−5.98	1	1.46	−9.11	−2.85	1.08
*B* ^2^	0.81	1	1.46	−2.32	3.94	1.08
*C* ^2^	−3.96	1	1.46	−7.09	−0.83	1.08
*D* ^2^	−0.52	1	1.46	−3.65	2.61	1.08

**Table 7:** Assessment of rate of degradation by measuring BOD, COD, % TPH, and TPC for the bacterial isolate *S. rhizophila (MP-1) *KX082814 for observed and predicted optimum conditions through RSM-BBD.

Test parameter	Observed optimum parameters through RSM-BBD	Validation of predicted optimum condition through RSM-BBD
TPC (cfu/ml)	4.8 × 10^−8^	6.7 × 10^−9^
TPH (%)	71.82%	79.53%
BOD (mg/L)	253.5	325.2
COD (mg/L)	816	835.4

**Table 8:** Validation of crude oil degradation by GC/MS analysis after treatment with *S. rhizophila *(PM-1) KX082814 on 15th day.

Major components of crude oil GC/MS				Major residual components of crude oil after treatment			
Peak	R. time	Linear & branched	% area	Peak	R. time	Linear & branched	% area
1	7.447	C10H22	2.19	1	22.97	C14H30	6.4
2	10.113	C11H24	3.18	2	25.20	C16H34	7.7
3	11.625	C16H33Cl	0.63	3	29.35	C16H34	8.29
4	12.877	C12H26	4.96	4	33.13	C16H34	7.53
5	15.592	C13H28	7.4	5	36.59	C16H34	5.85
6	19.728	C16H34	2.41	6	38.21	C16H34	4.2
7	20.682	C15H32	8.66	7	31.28	C21H44	8.02
8	23.035	C16H34	8.56	8	17.55	C8H17I	0.69
9	25.273	C20H42	8.41	9	41.28	C9H19I	1.77
10	27.392	C18H38	7.6	10	29.20	C11H24	1.43
11	29.413	C18H38	7.03	11	18.15	C12H26	2.66
12	34.932	C24H50	3.68	12	27.52	C12H26	4.61
13	36.617	C24H50	2.61	13	39.77	C13H28	3.23
14	38.234	C24H50	1.74	14	25.32	C20H42	10.23
15	39.790	C27H56	1.17	15	27.33	C20H42	7.13
16	41.291	C27H56	0.67	16	34.89	C20H42	6.89
17	42.739	C27H56	0.55	17	42.73	C6H14	1.01
18	44.137	C27H56	0.34	18	26.53	C11H24	0.64
19	45.485	C27H56	0.25	19	21.89	C12H26	0.63
20	46.792	C28H58	0.15	20	30.72	C12H26	0.71
21	48.057	C34H70	0.14				
22	8.046	C11H24	0.86				
23	11.743	C12H26	0.18				
24	11.859	C12H26	0.4				
25	13.242	C13H28	0.87				
26	14.287	C13H28	0.09				
27	14.336	C13H28	0.02				
28	24.106	C18H38	2.83				
29	25.390	C19H40	6.38				
30	27.561	C20H42	2.91				
